# Identification of Proximal and Distal 22q11.2 Microduplications among Patients with Cleft Lip and/or Palate: A Novel Inherited Atypical 0.6 Mb Duplication

**DOI:** 10.1155/2015/398063

**Published:** 2015-11-12

**Authors:** Maryam Sedghi, Hossein Abdali, Mehrdad Memarzadeh, Mansoor Salehi, Narges Nouri, Majid Hosseinzadeh, Nayereh Nouri

**Affiliations:** ^1^Medical Genetics Laboratory, Alzahra University Hospital, Isfahan University of Medical Sciences, Isfahan, Iran; ^2^Craniofacial and Cleft Research Center, Isfahan University of Medical Sciences, Isfahan, Iran; ^3^Department of Surgery, School of Medicine, Isfahan University of Medical Sciences, Isfahan, Iran; ^4^Pediatric Surgery Department, Imam Hossein Hospital, Isfahan University of Medical Sciences, Isfahan, Iran; ^5^Division of Genetics and Molecular Biology, Medical School, Isfahan University of Medical Sciences, Isfahan, Iran

## Abstract

Misalignments of low-copy repeats (LCRs) located in chromosome 22, particularly band 22q11.2, predispose to rearrangements. A variety of phenotypic features are associated with 22q11.2 microduplication syndrome which makes it challenging for the genetic counselors to recommend appropriate genetic assessment and counseling for the patients. In this study, multiplex ligation probe dependent amplification (MLPA) analysis was performed on 378 patients with cleft lip and/or palate to characterize rearrangements in patients suspected of 22q11.2 microduplication and microdeletion syndromes. Of 378 cases, 15 were diagnosed with a microdeletion with various sizes and 3 with duplications. For the first time in this study an atypical 0.6 Mb duplication is reported. Illustration of the phenotypes associated with the microduplications increases the knowledge of phenotypes reported in the literature.

## 1. Introduction

Rearrangements in 22q11.2 region result in different syndromes including 22q11.2 Cat-eye syndrome, microdeletion syndrome (OMIM#192430), and microduplication syndrome. The 22q11.2 microdeletion syndrome is characterized by the deletions of 22q11.2 which leads to a variety of phenotypes including velocardiofacial syndrome (VCFS, MIM 192430), DiGeorge syndrome (DGS, MIM 188400), and conotruncal anomaly face syndrome [1]. 22q11.2 microduplication syndrome results from reciprocal duplications in the region and individuals with this rearrangement have a wide range of phenotypes or may be without any symptom [2]. Deletion and duplication are the result of nonallelic homologous recombination (NAHR) mechanism mediated by eight low-copy number repeats (LCRs) identified on 22q11.2 region. It is predicted that NAHR in this region leads to an equal frequency of deletions and duplications [3]. However, the number of reported duplications is half of the deletions [2, 4, 5].

The incidence of 22q11.2 duplication in the population is not estimated precisely because many individuals with this rearrangement are without symptom [6–8]. Cognitive impairment and facial dysmorphism are the most common feature observed in more than half of the patients with duplications of 22q11 [1]. The other clinical features include velopharyngeal insufficiency, congenital heart disease, anal and urogenital abnormalities, congenital hypothyroidism, musculoskeletal problems, and ocular manifestations [1, 9–11]. 22q11.2 duplication may be inherited with an autosomal dominant pattern or occur because of a de novo rearrangement [1]. No correlation is found between the size of duplication and the severity of the phenotype in the patients. The size of the duplicated region depends on the chromosomal location of the LCRs involved in rearrangement [2].

The present study was conducted to evaluate the clinical signs of individuals with 22q11.2 duplication who were detected during assessing patients with cleft lip and/or palate by multiplex ligation-dependent probe amplification (MLPA) test. An elaborated description of the symptoms of the patients with this syndrome might help genetic counselors and pediatricians in providing better diagnosis of these patients.

## 2. Materials and Methods

### 2.1. Subjects

This study was approved by the ethics committee of the university board on human research and consent forms were obtained from all the patients or their parents. Blood samples were taken from 378 cases with cleft lip and/or palate admitted at cleft palate clinic at Medical University of Isfahan during a 5-year period (2006–2011). The patients include 213 males and 165 females, aged from 18 months to 27 years. DNA was extracted from blood samples using Qiagen DNA Mini kit (cat. number 51304) according to manufacturer's instructions. All DNA samples were quantified using NanoDrop instrument (Thermo 2000c).

### 2.2. MLPA Analysis

To determine copy number changes in 22q11.2 region, the SALSA MLPA kit P250-B1 DiGeorge (MRC-Holland, Amsterdam, Netherlands) was used. MLPA was performed according to manufacturer's recommendation. The P250 kit contains several probes for long arm of chromosome 22 and some probes for regions including 10p14 (DGS2), 4q35, 8p23, and 17p13 to identify other chromosomal abnormalities with features of DiGeorge anomaly that are not associated with 22q11 deletion. Amplified products were detected with ABI-3130xl (Genetic Analyzer). For MLPA analysis raw data were exported into GeneMarker software. A 35–50% increased or decreased relative peak area was taken as sign of heterozygous duplications or deletions in 22q11.2, respectively.

## 3. Results

Among all the patients with cleft lip and/or palate included in this study, 17 patients had rearrangements in 22q11.2. Further analysis of parents and siblings of the patients with rearrangements indicated one more duplication in one of the siblings. We found deletions with various sizes in 15 patients and duplications in three patients. Twelve patients had identical large deletion of about 3 Mb (LCR22-A to LCR22-D), two a 2.3 Mb deletion (LCR22-A to LCR22-C), and one a 1.5 Mb deletion (LCR22-A to LCR22-B).

Palatal findings in the patients with 22q11.2 deletion syndrome were as follows: submucous cleft palate and velopharyngeal incompetence (VPI) in 5 patients; soft cleft palate and cleft uvula in 3 patients; soft cleft palate in 2 patients; submucous cleft palate in 2 patients; soft cleft palate, submucous cleft palate, cleft uvula with asymmetric pharyngeal movement, and velopharyngeal incompetence (VPI) in one patient; submucous cleft palate with no movement of palate in one patient; hard cleft palate and submucous cleft in one patient.

In the following, a full description of three rare duplications found in this study including a 3 Mb and a 0.6 Mb duplication identified in a male and a female patient, respectively, and a reciprocal duplication of the LCRA-D detected in a 12-year-old girl is provided (Figure 2).

**Case 1 .**

**Figure 1:**
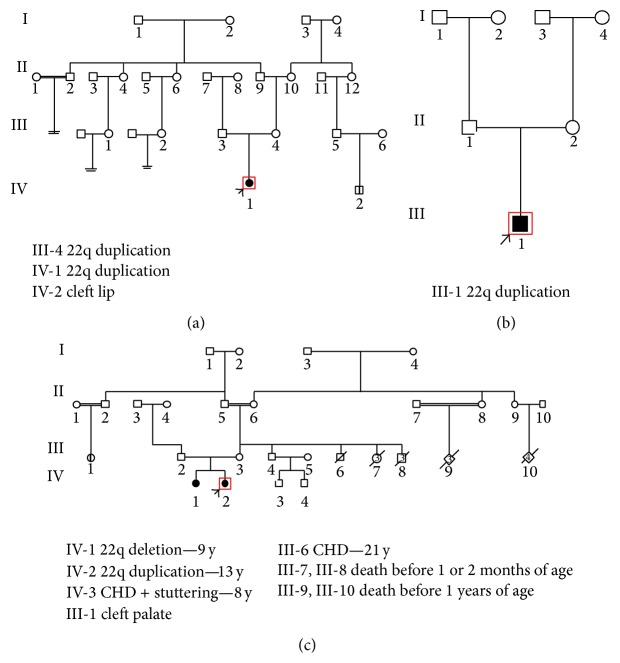
Family pedigree of 3 patients with clinical features of 22q11.2 duplication syndrome. (a) Case 1, (b) Case 2, and (c) Case 3.

**Figure 2:**
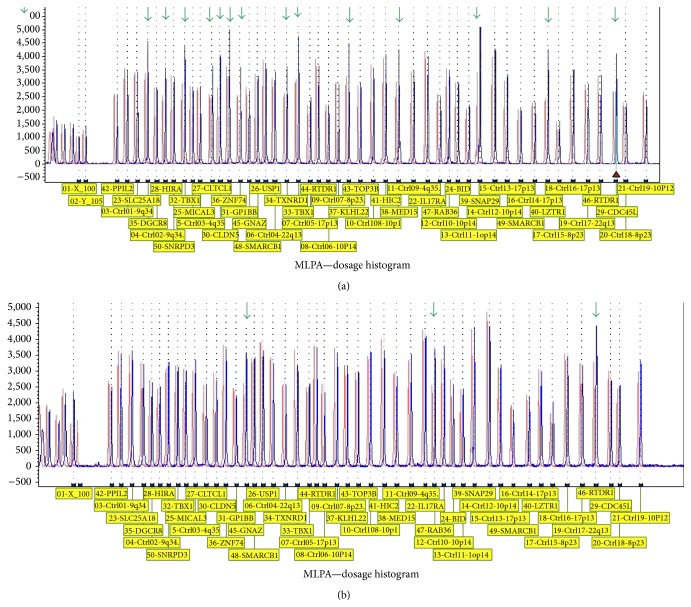
Multiplex ligation probe dependent amplification (MLPA) plots of our patients with clinical features of 22q11.2 duplication syndrome. Red spots show the duplication of probes located in 22q11.2 region. (a) Duplication of probes from GNAZ to RAB36 in Case 1 corresponding to distal duplication of TDR. (b) Duplication of probes from CLTC1 to LZTR1 in Cases 2 and 3 corresponding to reciprocal duplication of typically deleted region (TDR).

The patient was a 4-year-old girl (IV-1), the first child of a nonconsanguineous marriage with healthy parents and no family history of abnormalities except for one case of cleft lip in the grandchild of her mother's aunt (Figure 1(a)). She was born by elective cesarean section after 38 weeks of uneventful pregnancy with no history of maternal exposure to teratogens. During neonatal period, she had one convulsion at the 12th day and after 50 days it was repeated. At birth, she presented soft palate cleft. Her birth weight, length, and occipitofrontal circumference (OFC) were within normal range. Her APGAR scores were 9/1′′ and 10/5′′. No signs of cyanosis or early jaundice were observed. Her EEG was abnormal. Her echocardiogram and ultrasound of brain and abdomen and brain CT scan show no abnormal findings. Visual and hearing acuity were within normal limits. A soft cleft surgery was performed at 7th month. Her initial motor development was good. She walked at 12 months and started to say one word and make some sounds. At the age of 2.5 years, the parents noticed a subsequent arrest of development and the patient was referred to our center for speech delay at the age of 3. She was in good general health; weight, height, and OFC were within normal range but she was restless and unable to speak. She presented with narrow forehead, prominent nasal bridge, bilateral epicanthal fold, periorbital fullness, deep set eye, upslanting palpebral fissures and upward eyebrow, full cheeks, micrognathia, and small tapering fingers. At the age of 4, she had heat intolerance, repetitive behavior, velopharyngeal insufficiency, learning disability, hyperactivity, and attention deficit. Early intervention for speech improvement showed gradual but not remarkable progress, so fluoroscopy was done. Duplication of LCR 22E-F was identified after MLPA analysis (Figure 2(a)). Her mother had the same duplication but without symptom, so it seems that she inherited this duplication from her mother. No other history was mentioned in family pedigree.

**Case 2 .**

The patient was an 11-year-old boy (III-1), the first child of nonconsanguineous marriage. His parents are 37 and 44 years old with no history of hereditary disease in the pedigree; both parents were phenotypically normal (Figure 1(b)). His birth weight, length, and occipitofrontal circumference (OFC) were within normal range. His APGAR scores were 9/1′′ and 10/5′′. He had cleft palate (soft and hard) that was diagnosed at birth. His milestone development and learning ability were normal. When he was admitted to the genetic clinic, no dysmorphic feature was observed. Weight and length were 22 kg and 128 cm which revealed poor growth. The duplication of LCRA-D was detected in 22q11.2 region which was inherited from his father (Figure 2(b)).

**Case 3 .**

The third case was a 13-year-old girl (IV-2), the first child of a nonconsanguineous marriage with healthy parents. She had no dysmorphic features including any types of cleft lip or palate; therefore, no molecular analysis was performed until deletion of LCR22A-D was detected in her sister. A reciprocal duplication of LCR22A-D deletion was detected after MLPA analysis in the proband (Figure 2(b)). She was born by elective cesarean section after 39 weeks of uneventful pregnancy with birth weight of 1900 g, length 48 cm, and head circumference 31 cm which all were within 3–25th centile for the age of gestation. APGAR scores were 9/1′′ and 10/5′′. All of her developmental milestones were delayed. Head control was achieved at the age of 4 months, unsupported sitting at 8 months, and walking at 15 months. Her language acquisition was also delayed as she could make words at 2 years and she could not speak properly up to 7 years old. Visual and hearing acuity were within normal limits. Echocardiogram was done at her 6 months of age and revealed mild tricuspid regurgitation; however, after 3 years, the tricuspid valve function improved spontaneously. No VSD or PDA was observed. At age 12 when she and her sister were referred to genetic counselor, she only had mild learning disability. Interestingly, the pedigree of family revealed her maternal uncle had a congenital heart defect that in spite of operation had caused him to die at the age of 20. Her maternal grandmother had 7 newborn deaths and 2 aunts of her mother had 3-4 newborn deaths. In addition, her mother had a first cousin with cleft lip (Figure 1(c)). No deletion or duplication was detected in the parents.

## 4. Discussion

22q11.2 microduplication syndrome is a newly described syndrome with over 50 cases reported in the literature [4, 12]. The number of clinically described patients with 22q11.2 microduplication syndrome is increasing fast by the introduction of MLPA as a screening approach for these patients. The size of duplicated region reported in the literature extends from 479 kb to 6 Mb including the reciprocal duplication of TDR [1, 13, 14]. There is only one report on investigation of 22q11 region rearrangements in Iran where a duplication was identified in patients with mental retardation [15].

In this study, microduplications of 22q11.2 region were identified in 3 cases by MLPA method, 2 of whom were referred to medical genetics lab in a cohort study for identification of rearrangements in 22q11.2 in patients with cleft lip and/or palate. The third patient was characterized as a sibling of a proband with LCRA-D deletion. To confirm MLPA results, interphase FISH analysis was carried out on fixated whole blood samples from the probands and their parents.

The clinical phenotypes of the cases vary widely and apparently and as reported previously do not correlate with the size of duplication. In the present study, two cases, Cases 2 and 3, were identified with the LCR22A-D duplications. The only abnormality found in Case 2 was cleft palate and Case 3 had only mild learning disability when referred for clinical examination.  Case 1 carrying the LCRE-F duplication had a spectrum of clinical phenotypes which somewhat overlaps with the features of DGS/VCFS syndrome. Cases 1 and 2 had parents with the same duplicated region but with no abnormality in phenotype. In Case 3, no deletion or duplication was detected in the parents by MLPA and FISH methods. To determine the parental origin of the chromosomal rearrangements, SNP arrays should be performed on DNA from both parents and siblings.

The reports show that a majority of cases with 22q11 microduplication inherited the duplication from healthy or only minor affected parents. This wide phenotypic spectrum observed in different cases and their families may result from modifying factors including the interaction between mutations in genes not located in 22q11.2 region, other chromosomal anomalies, or environmental factors with 22q11.2 duplications [16, 17].

To our knowledge, LCRE-F duplication is reported for the first time in this study. The deletion of the same region was previously reported in a patient with heart disease and mild dysmorphic features by Rauch et al. [18]. Interestingly, our case with LCRE-F duplication had no heart abnormality; however, she showed severe dysmorphic features including soft cleft palate.

In our cleft palate population, 2 of 378 (3.97%) patients had 22q11 duplication syndrome while 15 of 378 patients were affected with 22q11.2 deletion. Lower frequency of 22q11.2 duplications compared with deletions was observed in our examined cleft palate population. Our present study shows that palatal anomalies are not probably a common feature in 22q11.2 duplication syndrome. Therefore, in controversy to 22q11.2 deletion, cleft palate may not be a very appropriate criterion for diagnosis of 22q11.2 duplication syndrome. Our result is somehow also in accordance with the work of Sivertsen et al. (2007) on 169 babies born in Norway with CPO during a 5-year period (1996–2001) that found no patient with 22q11.2 duplication [19].

Reports on new atypical microduplication will extend the knowledge on phenotypic spectrum of the syndrome which in turn will enable pediatricians and genetic counselors to provide better diagnosis and counseling of the patients' families. The confirmation of the diagnosis of this syndrome is only possible by the exact characterization of the rearrangement in 22q11.2 region. Among all the techniques introduced for detection of chromosomal rearrangements, MLPA technique is the most precise and fast which should be performed in all the cases suspicious of 22q11.2 rearrangements. Furthermore, array-CGH analysis for detecting the precise breakpoints of 22q11.2 region and other copy number variants in the whole genome should be performed.
